# RNA-Binding Proteins in Adipose Biology: From Mechanistic Understanding to Therapeutic Opportunities

**DOI:** 10.3390/ijms27020756

**Published:** 2026-01-12

**Authors:** Ghida Dairi, Maria Al Ibrahim, Saeed Al Mahri, Khalid Al-Regaiey, Shuja Shafi Malik, Sameer Mohammad

**Affiliations:** 1 Research, Development and Innovation Unit, Umm Al-Qura University, Makkah 21961, Saudi Arabia; gsdairi@uqu.edu.sa; 2 Experimental Medicine, King Abdullah International Medical Research Center, King Saud Bin Abdulaziz University for Health Sciences, Ministry of National Guard Health Affairs, Riyadh 11481, Saudi Arabia; alibrahimm@kaimrc.med.sa (M.A.I.); almahrisa@mngha.med.sa (S.A.M.); maliksh@kaimrc.med.sa (S.S.M.); 3 Physiology Department, College of Medicine, King Saud University, Riyadh 11362, Saudi Arabia; kalregaiey@ksu.edu.sa

**Keywords:** obesity, adipose tissue dysfunction, adipogenesis, insulin resistance, RNA-binding proteins, metabolic homeostasis, post-transcriptional regulation

## Abstract

Obesity, defined by excessive body fat accumulation, is strongly associated with dysfunction of adipose tissue, a major regulator of whole-body energy balance and metabolic health. Dysfunctional adipose tissue is characterized by altered adipokine secretion, impaired insulin sensitivity, and chronic low-grade inflammation, all of which contribute to obesity-related comorbidities such as type 2 diabetes, cardiovascular disease, and certain cancers. Understanding how obesity disrupts adipose tissue biology is essential for developing strategies to mitigate these metabolic risks. In recent years, RNA-binding proteins (RBPs) have emerged as important regulators of energy metabolism. By controlling post-transcriptional gene expression, RBPs influence RNA stability, localization, and translation, thereby shaping key cellular processes. Dysregulation of specific RBPs has been implicated in obesity and metabolic disorders, with several shown to affect adipogenesis, lipid handling, thermogenesis, and insulin sensitivity across different adipose depots. Their ability to direct the fate of transcripts involved in metabolic homeostasis positions RBPs as critical nodes linking adipose dysfunction to systemic disease. This review provides a mechanistic overview of RBP functions in adipose biology, highlights how their dysregulation can reinforce metabolic dysfunction, and identifies gaps and future directions for exploring RBPs and their RNA networks as potential therapeutic targets for obesity and related metabolic diseases.

## 1. Introduction

Obesity has emerged as a significant challenge to human health, affecting millions of individuals worldwide and leading to a myriad of associated health complications [1,2,3]. This condition, characterized by excessive body fat accumulation, is not merely a cosmetic concern but a serious medical issue that increases the risk of chronic diseases such as diabetes, heart disease, and certain types of cancer [4,5,6]. The rise in the prevalence of obesity can be attributed to a combination of factors, including sedentary lifestyles, poor dietary choices, and genetic predispositions [1,7,8,9]. Furthermore, the societal implications of obesity are profound, as it can lead to discrimination, mental health issues, and increased healthcare costs [10,11]. Addressing obesity requires a multifaceted approach that includes promoting healthier eating habits, encouraging physical activity, and implementing public health policies aimed at creating supportive environments for healthier living [12,13]. Adipose tissue is an essential component of the body that stores energy, provides insulation, and regulates body temperature [14,15,16]. It produces hormones that regulate appetite, immune function, and inflammation [14]. Adipose tissue protects vital organs and produces vitamin D. Its ability to store excess energy in triglycerides promotes metabolic homeostasis and overall health. Its dynamic nature allows it to adapt to changing energy requirements, which improves overall metabolic health. There are two main types of adipose tissue: white adipose tissue (WAT) and brown adipose tissue (BAT) [15,17]. WAT stores energy in the form of triglycerides, while BAT is involved in thermogenesis to generate heat [17,18]. Additionally, brown adipose tissue has a higher concentration of mitochondria compared to white adipose tissue, which allows it to generate heat more efficiently through thermogenesis. This is why brown adipose tissue is often referred to as “good fat” due to its ability to help regulate body temperature and potentially aid in weight loss [16,19,20]. Obesity can lead to significant dysfunction within adipose tissue. This dysfunction manifests in various ways, including altered metabolic processes, inflammation, and hormonal imbalance [21,22,23]. Adipose tissue, which serves not only as a fat storage depot but also as an active endocrine organ, can become resistant to insulin and disrupt the regulation of glucose and lipid metabolism. Additionally, BAT activity may be reduced in obese individuals, leading to a decrease in energy expenditure and potentially exacerbating weight gain. Overall, the imbalance between WAT and BAT in obese individuals can contribute to metabolic dysfunction and further weight gain [21]. Furthermore, the overabundance of adipose tissue can contribute to a chronic state of low-grade inflammation, which is linked to numerous health issues such as cardiovascular disease, type 2 diabetes, and certain cancers [24,25,26]. Understanding the complexity of excess weight and how it affects adipose function is essential for creating successful obesity-related healthcare interventions. As a result, understanding the specific processes governing adipose function as well as the molecular events that lead to adipose tissue dysfunction is crucial for developing targeted therapies to combat obesity and its associated health risks.

## 2. Regulatory Controls of Adipogenesis and Adipose Tissue Function

**Figure 1:**
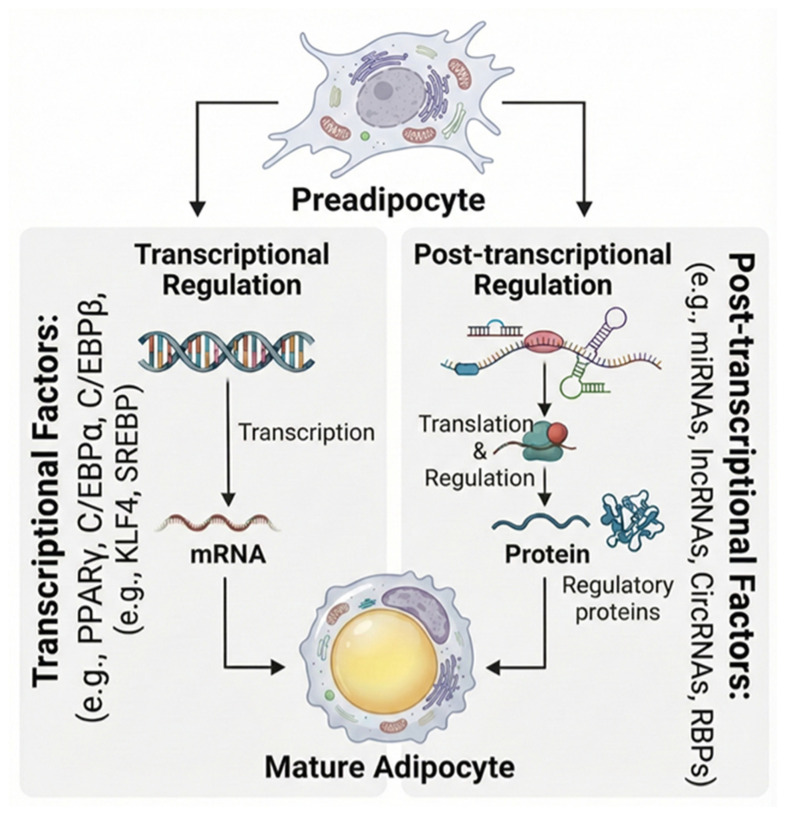
Transcriptional and post-transcriptional regulation of adipogenesis.

Adipose tissue is primarily composed of adipocytes, or fat cells, which serve as the body’s main energy reservoir by storing triglycerides. However, the functional significance of adipocytes extends far beyond passive fat storage [14,15,27]. These metabolically active cells play a central role in maintaining energy homeostasis and regulating systemic metabolism [15,28]. During periods of energy demand, stored triglycerides are mobilized to supply fuel to other tissues, ensuring metabolic balance. In addition, adipocytes act as endocrine cells by secreting adipokines, bioactive signaling molecules that modulate insulin sensitivity, inflammation, appetite regulation, and overall metabolic health [14,16]. Through these dynamic activities, adipose tissue contributes to maintaining body weight stability and protecting against metabolic disorders. Adipogenesis, is a highly coordinated process in which preadipocytes differentiate into mature lipid-storing fat cells [29,30,31]. This transformation is regulated by a complex network of transcription factors and signaling pathways that regulate the expression of genes involved in lipid metabolism, insulin responsiveness, and inflammatory control [30,32]. At the core of this transcriptional cascade lie peroxisome proliferator-activated receptor gamma (PPARγ) and members of the CCAAT/enhancer-binding protein (C/EBP) family (C/EBPβ and C/EBPδ) [33,34,35,36,37]. Together, they establish a stable and irreversible adipocyte identity by activating the full suite of genes necessary for metabolic function. Secondary transcriptional regulators, including Krüppel-like factors (KLFs) and Sterol Regulatory Element-Binding Proteins (SREBPs), further refine this program, fine-tuning lipid synthesis and coordinating the timing of differentiation [31,32]. In addition to these well-characterized transcriptional mechanisms, post-transcriptional regulation has emerged as a vital layer of control in adipogenesis [38,39]. Operating after mRNA synthesis, this regulatory dimension determines how transcripts are processed, stabilized, and translated, thereby shaping the ultimate protein output of adipocytes. Key post-transcriptional mechanisms include alternative splicing, which generates diverse mRNA isoforms from a single gene to expand proteomic diversity, and mRNA stability and decay, which govern transcript longevity in the cytoplasm. These processes are tightly orchestrated by RNA-binding proteins (RBPs) and non-coding RNAs, such as microRNAs and long non-coding RNAs, which interact with specific mRNA targets to modulate their splicing, localization, translation efficiency, and degradation [31,40,41,42,43]. Through these interactions, post-transcriptional regulators enable adipocytes to rapidly adjust gene expression in response to developmental cues, hormonal fluctuations, and metabolic stress. The factors involved in adipogenic differentiation are illustrated in Figure 1.

Beyond differentiation, both transcriptional and post-transcriptional regulation remain critical for maintaining adipose tissue function. PPARγ, for instance, continues to safeguard lipid metabolism and insulin sensitivity in mature adipocytes [42,44,45]. The transcriptional regulation of adipokines such as adiponectin, a hormone that enhances systemic insulin responsiveness, is also mediated by PPARγ and C/EBPα, underscoring the importance of transcriptional control in endocrine function [45,46,47]. Conversely, perturbations in this finely tuned regulatory network can lead to adipose tissue dysfunction. Repressors such as Pref-1 and GATA2/3 impede early adipogenesis, while reduced PPARγ activity or altered cofactor interactions in obesity contribute to impaired adipocyte function and insulin resistance [48,49,50,51]. Similarly, disruptions at the post-transcriptional level such as aberrant RBP activity or dysregulated miRNA expression can destabilize mRNA networks essential for metabolic homeostasis [43,52,53,54,55,56]. Overall, the functional integrity of adipose tissue is maintained by a multi-layered regulatory hierarchy. Transcriptional mechanisms, led by PPARγ and C/EBP family members, establish the foundational adipogenic and metabolic programs, while post-transcriptional processes act as adaptive modulators, fine-tuning gene expression in response to physiological and pathological cues. Disruptions at either regulatory level can unsettle this delicate equilibrium, transforming adipose tissue from a state of healthy, adaptive expansion into dysfunctional depots that promote insulin resistance and drive metabolic disease. Deepening our understanding of these interconnected transcriptional and post-transcriptional networks will be critical for uncovering novel therapeutic targets capable of restoring adipose tissue function and alleviating obesity-related metabolic disorders.

## 3. RNA-Binding Proteins in the Regulation of Adipogenesis and Adipose Metabolism

**Figure 2:**
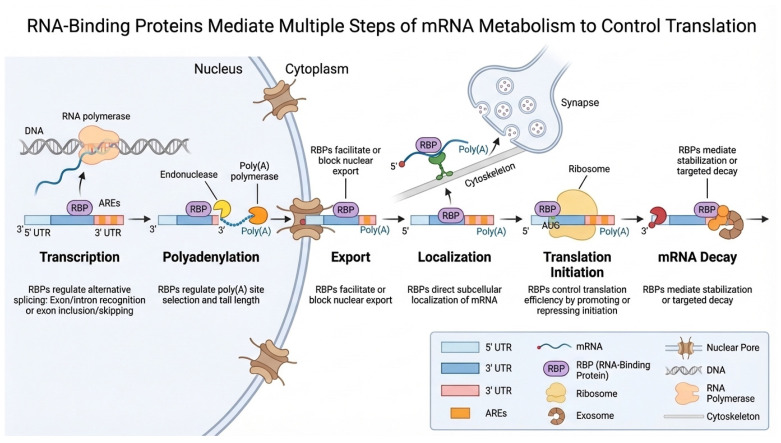
RNA-binding protein-mediated control of mRNA translation.

RNA-binding proteins (RBPs) are central regulators of post-transcriptional gene expression, acting at nearly every step of an RNA molecule’s life cycle [57]. Their functional importance stems from their ability to selectively recognize RNA sequences or structures and translate these interactions into precise regulatory outcomes (Figure 2).

By controlling RNA splicing, polyadenylation, export, localization, translation, and decay, RBPs shape both the amount and the timing of protein production in cells [58,59]. Many RBPs work in dynamic, combinatorial networks, allowing cells to rapidly reprogram gene expression in response to developmental cues, metabolic demands, or environmental stress. Beyond their canonical roles, RBPs also influence RNA modifications, ribonucleoprotein assembly, and phase-separated condensates that organize biochemical reactions in space and time. Dysregulation of RBPs through genetic mutations, altered expression, or pathogenic mislocalization disrupts RNA homeostasis and can drive a wide spectrum of diseases, including neurodegeneration, cancer, metabolic disorders, and viral pathogenesis [60,61,62,63,64,65] Collectively, RBPs provide a highly adaptable and multilayered regulatory system that ensures fidelity, flexibility, and context-specific control of gene expression. Recent studies have underscored the importance of RBPs in the functionality of adipose tissue and their contribution to the dysfunction associated with obesity. A number of these proteins have been thoroughly investigated regarding their roles in the differentiation of adipocytes, the metabolism of lipids, and the sensitivity to insulin. RBPs play a crucial role in regulating lipid storage and maintaining energy balance, which influences the body’s capacity to store fat and its reaction to dietary signals. Additionally, the improper regulation of RBPs has been associated with obesity and various metabolic disorders, emphasizing their critical role in preserving the health of adipose tissue and ensuring overall metabolic equilibrium. A summary of RBPs known to play key roles in these processes is presented in Table 1, and their specific functions are discussed in detail in the following sections.

### 3.1. Quaking Protein (QKI)

The QKI protein, or Quaking protein, is a member of the STAR (signal transduction and activation of RNA) family of RNA-binding proteins, which play a crucial role in the regulation of gene expression at the post-transcriptional level [104,105,106]. This protein is characterized by the presence of multiple K-homology (KH) domains, which facilitate its interaction with RNA molecules [107,108]. QKI is particularly significant in the development of the nervous system, where it influences the differentiation of oligodendrocytes and the formation of myelin sheaths, essential for proper neuronal function. QKI also plays important roles in cardiac function, smooth muscle differentiation, and monocyte-to-macrophage differentiation [104,105,109,110]. Additionally, QKI has been implicated in the regulation of cell proliferation and apoptosis, highlighting its importance in maintaining cellular homeostasis. Its dysregulation has been associated with several diseases, including cancer and neurological disorders, making it a vital focus of research in understanding these conditions and developing potential therapeutic strategies [105,106,109,111,112]. Recent studies have outlined the importance of QKI in glucose and lipid metabolic homeostasis. First, genetic variants within the QKI locus have been documented to portray genome-wide significance for height, BMI, T2D, total cholesterol (TC), low-density lipoprotein (LDL), high-density lipoprotein (HDL), and triglyceride (TG) levels [113]. In addition, QKI variants have been found to exhibit significant binding sites for well-known transcription factors, especially in human subcutaneous adipose tissue [114,115]. A pivotal study by Huanyu Lu et al. demonstrated that QKI expression is significantly upregulated in both brown adipose tissue (BAT) and white adipose tissue (WAT) of mice subjected to a high-fat diet (HFD) [116]. This finding positioned QKI as a potential key mediator in the body’s response to nutritional stress. To elucidate the functional consequences of this upregulation, they generated adipose-specific QKI-knockout mice. These knockout models exhibited a striking resistance to diet-induced obesity. Phenotypically, this was characterized by a lower overall fat mass, a reduction in adipocyte size, and diminished lipid droplet accumulation within adipocytes compared to their control counterparts. The metabolic benefits extended beyond mere weight control; QKI-deficient mice also demonstrated enhanced glucose tolerance, indicating improved systemic metabolic health. Furthermore, the ablation of QKI had protective effects on other organs, notably reducing ectopic fat deposition in the liver and attenuating the chronic, low-grade inflammation hallmark of obesity, as evidenced by decreased inflammatory cell infiltration into adipose tissue. The study revealed that QKI is activated by intracellular cAMP signaling, a central pathway in metabolic regulation. Once activated, QKI exerts post-transcriptional control by translationally regulating key thermogenic and metabolic genes. Specifically, it represses the synthesis of Uncoupling Protein 1 (UCP-1), which is responsible for non-shivering thermogenesis in BAT, and peroxisome proliferator-activated receptor gamma coactivator 1-alpha (PGC-1α), a master regulator of mitochondrial biogenesis and energy expenditure. Through this dual regulation, QKI acts as a molecular switch that negatively modulates BAT activity and overall energy balance [116]. While these findings are compelling, the relevance of these findings to human physiology is currently unknown. It remains to be established whether QKI is expressed in human BAT and WAT and how its expression and functional activity are modulated in the adipocytes of individuals with obesity. Investigating the QKI pathway in human adipose tissue samples across a spectrum of body mass indices could validate its role as a conserved therapeutic target. In conclusion, the current evidence strongly suggests that QKI is a druggable node linking dietary stress to adipose tissue dysfunction. Pharmacological inhibition of QKI could therefore represent a novel and attractive therapeutic strategy to combat obesity and its wide-ranging metabolic complications, including insulin resistance and fatty liver disease.

### 3.2. HuR (Human Antigen R)

**Figure 3:**
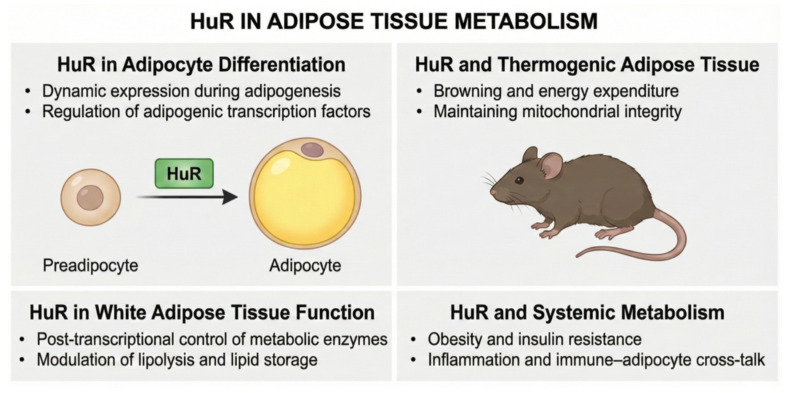
Functional significance of HuR in adipose tissue metabolism.

Human antigen R (HuR), also known as ELAV-like protein 1, is a member of the ELAV (embryonic lethal abnormal vision) protein family, which plays a crucial role in post-transcriptional regulation of gene expression [66,117]. HuR is encoded by the ELAVL1 gene in humans and is widely expressed in a variety of tissues, including endocrine organs, the respiratory system, and the gastrointestinal tract [118,119,120]. HuR is primarily found in the nucleus, where it participates in pre-mRNA splicing and nuclear export of mature mRNAs. In response to specific stimuli, HuR can translocate to the cytoplasm, where it binds to AU-rich elements (AREs) in the 3′ untranslated regions of target mRNAs. By stabilizing these mRNAs, HuR enhances their translation, thereby influencing various cellular processes such as proliferation, differentiation, and stress responses. Furthermore, HuR has been implicated in several pathological conditions, including cancer, where its overexpression is often associated with tumor progression and poor prognosis [118,119,120,121,122,123,124,125]. The multifaceted functions of HuR underscore its significance in both normal physiology and disease, making it a potential target for therapeutic interventions aimed at modulating its activity or expression. Recent studies increasingly highlight HuR as a key regulator of adipogenesis and adipose tissue function. Indeed, among RNA-binding proteins, HuR remains the most extensively studied in the context of adipose metabolism [126,127,128,129]. HuR expression is negatively associated with obesity in both mice and humans. In comparison to healthy controls, HuR expression is significantly reduced in subcutaneous adipose tissue of obese human subjects. Similarly, its expression is markedly decreased in WAT and BAT from animal models of obesity and type 2 diabetes, as compared with their controls. Gantt et al. demonstrated that HuR plays a key role in the onset of adipogenic differentiation of 3T3-L1 cells [130]. In 3T3-L1 cells, HuR is predominantly localized in the nucleus but translocates to the cytosol after incubation with a differentiation cocktail, consistent with HuR regulating the availability of relevant mRNAs for translation. HuR knockdown attenuated the differentiation process, highlighting the importance of HuR in adipogenic differentiation. Using adipose-specific HuR-knockout (HuR-KO) mice, Jingyuan Li et al. showed that HuR-KO mice gained more weight and had higher fat mass when challenged with a high-fat diet [128]. After 6 months on an HFD, HuR-KO mice had significantly greater fat mass compared to control mice. Additionally, HuR-KO mice had higher serum levels of total cholesterol, triglycerides, and low-density lipoprotein (LDL) and lower levels of high-density lipoprotein (HDL). These findings suggest that HUR deficiency predisposes mice to HFD-induced obesity and lipid metabolism disorders. Further research revealed that HuR-KO adipocytes were hypertrophic and had more lipid accumulation due to inhibition of lipolysis. HUR regulates the protein expression of adipocyte triglyceride lipase (ATGL) by stabilizing its mRNA. ATGL is an enzyme that catalyzes the hydrolysis of triglycerides into free fatty acids and glycerol, allowing stored fat to be mobilized for energy generation. Its function is critical for maintaining energy homeostasis, especially during times of fasting or elevated energy demand. ATGL also plays an important function in controlling lipid droplet dynamics and contributing to the overall balance of lipid storage and utilization in adipose tissue and other cells. ATGL levels are lower in obese human adipose tissue, which coincides with lower HuR expression, implying a possible relationship between HuR and ATGL dysregulation in obesity. The authors concluded that adipose HuR regulates lipid breakdown through ATGL protein translation, thereby controlling obesity and metabolic syndrome. A subsequent study by Siang et.al confirmed the involvement of HuR in adipose functioning and, consistent with earlier observation, adipose-specific HUR-KO mice exhibited significantly increased fat mass, along with glucose intolerance and insulin resistance [129]. HuR was found to be a repressor of adipogenesis in both white adipose tissue (WAT) and brown adipose tissue (BAT). HuR knockdown in brown and white preadipocytes resulted in enhanced expression of pan-adipocyte markers and lipid accumulation. On the other hand, HuR overexpression dramatically reduced lipid accumulation and adipogenic marker expression indicating that HuR has a suppressive role in adipogenesis. HuR controls adipogenesis by regulating the mRNA stability and translation of insulin-induced gene 1 (INSIG1), which is a known regulator of lipogenesis and lipid homeostasis. Another study found that HuR expression in adipocytes is essential for maintaining metabolic homoeostasis and mediates BAT thermogenesis that is independent of UCP1 via post-transcriptional regulation of intracellular calcium transport [131]. The same group also showed that adipocyte HuR also played a key role in maintaining cardiac health, and adipocyte-specific deletion of HuR induced spontaneous cardiac hypertrophy and fibrosis [67]. These findings suggest that HuR plays a crucial role in regulating various metabolic processes beyond adipogenesis, including lipid homeostasis and cardiac health. A recent study has renewed debate over the function of HuR in adipose tissue. Fan et al. found that adipocyte-specific HuR deletion protected mice from high-fat diet-induced weight gain by promoting adipose browning and thermogenesis, marked by increased Ucp1 expression and higher energy expenditure [68]. They propose that HuR normally suppresses Ucp1 by binding its 3′UTR, so loss of HuR releases this repression and activates thermogenic pathways. The study also reported enhanced cytoplasmic localization of HuR in obesity and highlighted how its results contrast with earlier studies in which HuR deletion caused, rather than prevented, diet-induced obesity. According to the authors, these conflicting outcomes likely arise from differences in HuR target engagement (e.g., ATGL vs. Ucp1), variations in dietary regimens and experimental design, and the distinct physiological endpoints evaluated across studies. The functional significance of HuR in adipose tissue metabolism is summarized in Figure 3.

Future research should aim to resolve this debate over the in vivo function of HuR in adipose tissue by using standardized knockdown or overexpression strategies and carefully controlled environmental conditions. Human validation will be essential, including analyses of HuR expression, subcellular localization, and target mRNA levels in well-phenotyped adipose biopsies (both subcutaneous and visceral) from large cohorts with detailed metabolic profiling; longitudinal sampling, such as before and after weight loss, bariatric surgery, or cold exposure, would add valuable insight. In parallel, small-molecule modulation of HuR, using emerging inhibitors from oncology or compounds that alter HuR–RNA binding or trafficking, could help determine whether targeting HuR yields beneficial metabolic effects and whether these actions are depot-specific or context-dependent.

### 3.3. Y-Box Binding Proteins

Y-box binding proteins (YBXs) are multifunctional nucleic-acid-binding proteins initially characterized by their ability to recognize the Y-box motif in DNA [69]. Structurally, they contain a central cold-shock domain (CSD), a highly conserved β-barrel domain associated with stress responses, flanked by an N-terminal alanine/proline-rich (A/P) domain and a disordered C-terminal region. While historically regarded as transcription factors, YBXs are now recognized as major RNA-binding proteins that influence mRNA stability, translation, and localization [69,70,71,72]. Both the CSD and the C-terminal domain contribute to RNA-binding capacity, allowing YBXs to regulate a broad array of transcripts involved in metabolic and developmental processes. Among the YBX family, YBX1 and YBX2 have emerged as key post-transcriptional regulators of lipid metabolism and adipose tissue homeostasis. YBX1 expression is upregulated during adipocyte differentiation, and its depletion suppresses lipid accumulation and the induction of adipogenic genes [132]. Mechanistically, YBX1 facilitates autophagy, a process essential for lipid droplet formation and adipocyte maturation. It promotes the expression of the autophagy-initiating kinases ULK1 and ULK2 through two complementary mechanisms: post-transcriptionally stabilizing Ulk1 mRNA via direct interaction with an m^5^C-modified site in its 5′ untranslated region and transcriptionally activating Ulk2 by binding to its promoter. These coordinated regulatory actions enhance autophagic flux, thereby supporting adipocyte differentiation and lipid storage. In vivo, overexpression of YBX1 increases white adipose tissue (WAT) mass, elevates autophagy markers, and upregulates ULK1/2 levels in response to a high-fat diet, underscoring its role in metabolic adaptation. YBX1 is also implicated in the regulation of insulin signaling in adipocytes [133]. YBX1 enhances the expression of the Ptp1b gene, which encodes the tyrosine phosphatase PTP1B. Because PTP1B dephosphorylates the insulin receptor (IR) on key tyrosine residues, elevated PTP1B levels dampen IR activation. Through this regulatory axis, YBX1 plays a key role in regulating adipocyte homeostasis and whole-body insulin sensitivity. Beyond its established role in white adipose tissue, YBX1 also plays a central part in thermogenic programming in both brown and beige adipocytes [134,135]. Rabiee et al. reported that cold exposure robustly induces YBX1 expression in brown adipose tissue (BAT) and that loss of YBX1 impairs preadipocyte differentiation and thermogenic capacity [134]. Conversely, enforced YBX1 expression during adipogenesis enhances mitochondrial respiration, upregulates thermogenic gene expression, and promotes the browning of white adipocytes. Transcriptomic profiling identified the histone demethylase JMJD1C as a key downstream effector, linking YBX1 to epigenetic control of thermogenic differentiation. Complementing these findings, Wu et al. demonstrated that YBX1 is highly expressed in BAT and further induced by cold and β-adrenergic stimulation [135]. Their loss-of-function studies showed that YBX1 deficiency disrupts brown adipocyte differentiation and suppresses thermogenic function. Mechanistically, YBX1 drives thermogenesis by promoting PINK1/PRKN-dependent mitophagy. RNA immunoprecipitation revealed that YBX1 directly binds *Pink1* and *Prkn* transcripts, while RNA decay assays showed that YBX1 is required for maintaining their mRNA stability. Consequently, YBX1 loss reduces PINK1 and PRKN protein levels, weakens mitophagy, and diminishes the thermogenic program. Importantly, in vivo overexpression of YBX1 in BAT enhances mitophagy and thermogenesis, confirming its functional relevance.

YBX2 also plays an essential role in brown adipogenesis and thermogenic activation [73]. Loss-of-function studies show that YBX2 knockdown severely impairs brown adipocyte differentiation, whereas its overexpression enhances BAT marker expression in both brown and white adipocytes. Although YBX2-knockout mice can form BAT, they fail to activate a full thermogenic gene program. Integrative RNA-sequencing and RNA immunoprecipitation analyses further identified a set of YBX2-bound mRNAs, including *Pgc1α* (peroxisome proliferator-activated receptor gamma coactivator 1-alpha), that become destabilized when YBX2 is depleted during cold exposure. PGC1α is a transcriptional coactivator that integrates environmental, hormonal, and metabolic cues to orchestrate major energy-metabolic programs. Working in concert with diverse transcription factors, it stimulates mitochondrial biogenesis, boosts oxidative phosphorylation, enhances fatty acid oxidation, and activates the thermogenic gene program that underpins brown and beige adipocyte function. A follow-up study demonstrated that the protein levels of YBX2 in adipocytes rise rapidly in response to thermogenic stimuli like cold or β3-adrenergic activation [74]. This accumulation is regulated post-transcriptionally through phosphorylation by AMPK or Akt2, which inhibits YBX2’s ubiquitination and degradation. Once stabilized, YBX2 enhances thermogenic capacity in two ways: first, by known mechanisms like stabilizing *Pgc1*α mRNA, and second, by a newly identified role in promoting glycolysis. YBX2 binds directly to the 5′-UTRs of glycolytic enzymes to increase their translation, thereby elevating glucose consumption and lactate output. These findings position YBX2 as a central, stimulus-responsive node that integrates signaling to fuel both glycolysis and thermogenesis in brown fat.

### 3.4. CELF1 (CUG-Binding Protein 1)

CELF1 (CUG-Binding Protein 1) is a ubiquitously expressed RNA-binding protein that functions as a master regulator of post-transcriptional gene expression [75]. CELF1 contains three RNA-recognition motifs (RRMs), two at the N-terminus (RRM1 and RRM2) that form a high-affinity tandem RNA-binding unit and a third (RRM3) near the C-terminus that adds additional binding strength. These domains enable CELF1 to specifically recognize UG-rich and CUG-repeat RNA sequences, allowing it to regulate alternative splicing, mRNA stability, deadenylation, and translation [75,76,77]. Through these diverse mechanisms, CELF1 exerts a powerful influence on a wide range of physiological and pathological states, with emerging evidence highlighting its indispensable role in adipose tissue function and systemic metabolic health. Population genetic studies have established a direct link between variations in the CELF1 gene locus and an increased risk of obesity, positioning it as a key player in the disease’s pathophysiology [78]. At the molecular level, CELF1 acts as an inhibitor of adipogenesis [79], and this inhibitory function becomes more pronounced with age. The mechanism involves CELF1 binding to the 3′ untranslated region (3′UTR) of the mRNA encoding *C/EBPβ*, a critical early transcription factor that drives adipocyte differentiation. By inhibiting the translation of *C/EBPβ* mRNA, CELF1 serves as a crucial checkpoint controlling adipose tissue expansion and homeostasis. Beyond its role in fat cell development, recent research has identified CELF1 as a physiological regulator of metabolic stress, specifically in activating thermogenesis to promote energy expenditure [80]. Multiple levels of experimental evidence solidified this role. First, CELF1 expression is significantly reduced in subcutaneous fat of individuals with obesity and negatively correlates with BMI. Second, CELF1 expression is significantly upregulated in white adipose tissue (WAT) upon thermogenic activation, such as exposure to cold temperatures or treatment with beta-adrenergic agonists. Third, genetically engineered mice lacking the Celf1 gene specifically in their adipocytes (CELF1 AKO mice) were unable to maintain their core body temperature in the cold. These mice exhibited a severe defect in adaptive thermogenesis, proving that CELF1 is functionally required for this heat production. Finally, overexpressing CELF1 in white fat cells was sufficient to induce beiging even at thermoneutral temperatures where this process does not normally occur. CELF1 regulates the thermogenic process by binding to and stabilizing the mRNA for Type 2 Iodothyronine Deiodinase (DIO2). The DIO2 enzyme is crucial because it locally converts the inactive thyroid hormone thyroxine (T4) into the active form, triiodothyronine (T3), which is a potent driver of the entire thermogenic program. Thus, through the specific stabilization of DIO2 mRNA, CELF1 sits at the nexus of a post-transcriptional pathway that is essential for adaptive thermogenesis and energy expenditure.

### 3.5. IGF2BP1 (Insulin-like Growth Factor 2 mRNA-Binding Protein 1)

IGF2BP1 is a pivotal RNA-binding protein that functions as a master regulator of gene expression at the post-transcriptional level. IGF2BP1 is a regulatory protein critical for embryonic development, controlling mRNAs involved in growth and organ formation [136,137,138,139]. While its expression is low in most adult tissues, it re-emerges in numerous cancers (e.g., lung, breast, liver). In this cancer context, IGF2BP1 functions as a powerful oncogene. It promotes tumorigenesis by stabilizing messenger RNAs that encode factors driving cell proliferation, inhibiting cell death, and enhancing metastasis, invasion, and resistance to chemotherapy [140,141,142,143]. Recent studies indicate possible involvement of IGF2BP1 in the regulation of adipose tissue biology and systemic metabolic health. A large-scale GWAS by Yingchang Lu et al., involving data from hundreds of thousands of individuals, identified specific SNPs associated with body fat percentage [81]. Notably, genetic variants linked to lower circulating IGF2BP1 levels were strongly associated with higher body fat percentage, independent of BMI. This suggests that, unlike many BMI-associated loci that predominantly implicate central nervous system pathways, these variants point to peripheral mechanisms, particularly adipocyte metabolism, as key contributors to adiposity. Complementing these findings, a genome-wide DNA methylation analysis identified IGF2BP1 as a major epigenetic marker connected to insulin sensitivity [82]. Comparing visceral adipose tissue (VAT) from insulin-resistant (IR) versus insulin-sensitive (IS) obese individuals, the study found significantly higher methylation of the IGF2BP1 gene in the IR group. Such hypermethylation likely suppresses IGF2BP1 expression. Given that IGF2BP1 is an RNA-binding protein that stabilizes transcripts essential for adipocyte homeostasis, its reduced expression may impair adipocyte function, promote inflammation, and exacerbate systemic insulin resistance. A recent study showed that the detrimental effect of estrogen supplementation on visceral adipose tissue in aged postmenopausal subjects is partly mediated by the activation of IGF2BP1 [83]. Elevated IGF2BP1 levels disrupt adipocyte homeostasis by altering the stability and translation of key metabolic transcripts, which serves to amplify estrogen’s negative consequences. Together, these findings point to IGF2BP1 as a key regulator of adipose tissue health, potentially influencing adipocyte differentiation, metabolic gene expression, and inflammatory signaling. They also highlight the possibility that disruptions in IGF2BP1-mediated pathways contribute to obesity-related adipose dysfunction. Future studies should investigate tissue-specific IGF2BP1 expression, delineate its molecular targets in adipocytes, and determine its causal role in adipose metabolism and systemic metabolic regulation.

### 3.6. ZFP36 (Zinc Finger Protein 36 Homolog)

ZFP36, commonly known as Tristetraprolin (TTP), is a critical RNA-binding protein that functions as a central post-transcriptional regulator of gene expression. It exerts its effects by binding to Adenylate/Uridylate-Rich Elements (AREs) within the 3′ untranslated regions (3′UTRs) of target messenger RNAs (mRNAs), leading to mRNA destabilization and degradation [144]. Through this mechanism, ZFP36 acts as a powerful brake on the production of a wide array of proteins, playing an indispensable role in maintaining cellular and systemic homeostasis, with profound implications in development and disease. The well-characterized function of ZFP36 is its potent anti-inflammatory activity [145,146]. It serves as a crucial negative feedback regulator, particularly for potent cytokines like Tumor Necrosis Factor-alpha (TNF-α). This role is dramatically illustrated in ZFP36-deficient mice, which develop a severe systemic inflammatory syndrome characterized by cachexia, destructive arthritis, and autoimmunity, driven primarily by the unchecked overproduction of TNF-α protein. Beyond individual cytokines, the ZFP36 family of proteins, as demonstrated by Cook et al., plays a critical and often redundant role in immune cell regulation, such as maintaining T cell homeostasis and preventing aberrant immune activation [147]. Beyond immunology, ZFP36 is increasingly recognized as a key modulator of metabolism. Evidence from human genetics identifies ZFP36 as a promising candidate gene for obesity-related metabolic complications. Polymorphisms in ZFP36 are associated with insulin resistance and dyslipidemia in obese individuals, suggesting a fundamental link between its function and metabolic health. In addition, elevated levels of the ZFP36 in visceral fat are linked to a healthier metabolic state [84]. Women with higher ZFP36 expression demonstrated improved insulin sensitivity, marked by lower fasting insulin, reduced insulin resistance (HOMA-IR), and lower insulin after glucose intake. This beneficial profile was further supported by a corresponding increase in adiponectin, a key insulin-sensitizing hormone. Together, these findings indicate that high ZFP36 in visceral fat is a marker of healthy adipose tissue and better whole-body insulin function. A recent study highlighted the role of ZFP36 in regulating adipose tissue metabolism [85]. ZFP36 expression is markedly reduced in adipose tissue from obese humans and in multiple obese mouse models (diet-induced, ob/ob, db/db). Functionally, mice lacking ZFP36 specifically in adipose tissue (ZFP36^AKO) and fed a high-fat diet for 16 weeks gained more weight and exhibited pronounced glucose intolerance and insulin resistance compared with controls. Their adipocytes were enlarged and showed reduced levels of key lipolytic proteins (PLIN1, ATGL, and HSL), indicating impaired lipid mobilization and excessive fat accumulation. Mechanistically, ZFP36 directly binds and destabilizes E3 ubiquitin-protein ligase RNF128 (*RNF128*) mRNA, suppressing its translation. Because RNF128 inhibits Sirtuin 1 (Sirt1), loss of ZFP36 increases RNF128 abundance, reduces Sirt1 activity, and drives adipose metabolic dysfunction. Overall, adipose ZFP36 emerges as a protective post-transcriptional regulator that limits adipocyte expansion and insulin resistance through the RNF128–Sirt1 axis, and its downregulation in obesity contributes to worsening metabolic outcomes. Future studies should focus on further elucidating the downstream effects of dysregulated lipolysis in the context of obesity-related pathologies, as well as exploring potential therapeutic interventions targeting the ZFP36/RNF128/Sirt1 pathway to mitigate metabolic dysfunction. A primary therapeutic goal would be to investigate strategies for restoring ZFP36 function. Future studies could explore the efficacy of gene therapy or small-molecule agonists designed to upregulate or mimic ZFP36 activity in adipose tissue, testing whether such interventions can reverse or prevent obesity and insulin resistance in preclinical models. Concurrently, the mechanistic pathway requires further elucidation; it would be crucial to determine if the metabolic benefits of ZFP36 are exclusively mediated through RNF128 degradation. This could be tested by generating and studying double-knockout mice (lacking both ZFP36 and RNF128 in fat cells) to see if depleting RNF128 rescues the metabolic defects caused by ZFP36 loss. Furthermore, the upstream regulation of ZFP36 remains an open question: what signals, such as hormones from other organs or nutrients, control its expression and activity, and why is it downregulated in obesity?

### 3.7. CPEB4 (Cytoplasmic Polyadenylation Element Binding Protein 4)

CPEB4 is an RNA-binding protein that plays a critical role in regulating gene expression at the level of translation. The core function of CPEB4 revolves around a specific sequence in the mRNA called the cytoplasmic polyadenylation element (CPE) [148,149]. CPEB4 binds to this CPE sequence in the 3′ untranslated region (3′ UTR) of its target mRNAs. Its primary mechanism involves controlling the length of the poly(A) tail, a string of adenine nucleotides at the end of an mRNA. The length of this tail is directly correlated with the mRNA’s translational efficiency: a long tail promotes efficient translation, while a short tail represses it. It plays a crucial role in neuronal plasticity and memory, regulating local protein synthesis at synapses and ensuring proper cell differentiation and growth [150,151,152]. Dysfunction of CPEB4 is linked to Autism Spectrum Disorder (ASD), as it disrupts the delicate balance of protein synthesis needed for proper brain wiring and function [153,154]. In cancer, CPEB4 is often overexpressed and acts as an oncogene, promoting the translation of proteins that drive tumor growth, blood vessel formation (angiogenesis), and metastasis [155,156,157]. In summary, CPEB4 is a critical molecular switch for protein synthesis, essential for brain function and development, but when dysregulated, it becomes a key player in diseases like cancer and autism. Emerging evidence from multiple studies has established cytoplasmic polyadenylation element binding protein 4 (CPEB4) as a critical regulator of adipose tissue biology, with its dysregulation being a significant contributor to metabolic dysfunction. The importance of this protein was first highlighted by human genetics. Genome-wide association studies have identified specific single-nucleotide polymorphisms (SNPs) near the CPEB4 gene that show a strong statistical association with key metabolic traits, particularly elevated waist-to-hip ratio (WHR) and body mass index (BMI)—two major indicators of obesity. Intriguingly, this same genetic locus was also linked to lower fasting glucose levels, an effect that was independent of its impact on body weight and shape, suggesting CPEB4 may have a direct role in glucose metabolism [86]. This genetic link is powerfully reinforced by observations of CPEB4 expression in both humans and animal models. In obese human subjects, the CPEB4 protein is significantly elevated in visceral white adipose tissue compared to that of non-obese individuals. This pattern is recapitulated in animal models of obesity, where CPEB4 expression is consistently upregulated in various adipose tissue depots [87]. To move from correlation to causation, researchers have employed genetic knockout models. When the Cpeb4 gene is deleted in mice, the animals are strikingly protected from high-fat-diet-induced weight gain. These knockout mice exhibit a marked reduction in adipose tissue enlargement and lower levels of tissue inflammation, a key driver of obesity-related metabolic disease. Furthermore, the absence of CPEB4 had a beneficial effect on the gut microbiome, attenuating the harmful dysbiosis typically caused by a high-fat diet and promoting a shift toward a more balanced and health-associated microbial profile. The mechanism underlying these systemic effects appears to be CPEB4’s fundamental role in driving the formation of new fat cells, a process known as adipogenesis. During the adipogenic differentiation of primary preadipocytes and the commonly used 3T3-L1 cell line, CPEB4 is highly upregulated. Functional studies demonstrate that knocking down CPEB4 reduces, in a cell-autonomous manner, the ability of these cells to differentiate into mature adipocytes and accumulate lipid droplets. This indicates that CPEB4 is not merely a bystander but is an essential molecular switch for fat cell development. In summary, the collective data paint a compelling picture: genetic variations in CPEB4 predispose individuals to obesity-related traits, while the protein itself is overexpressed in adipose tissue in obese states. It functions as a critical pro-adipogenic factor at the cellular level, and its inhibition, as shown in knockout models, can protect against diet-induced obesity, inflammation, and associated metabolic dysregulation, positioning CPEB4 as a potential therapeutic target for metabolic diseases.

### 3.8. HNRNPA1 (Heterogeneous Nuclear Ribonucleoprotein A1)

Heterogeneous nuclear ribonucleoprotein A1 (HNRNPA1) is the most abundant and ubiquitously expressed member of its family. Its RNA-binding ability is primarily mediated by two N-terminal RNA recognition motifs (RRMs) and a C-terminal low-complexity/RGG-rich region. It plays important roles in telomere maintenance and the biogenesis of certain microRNAs [158,159,160]. Due to its central role in RNA metabolism, dysregulation of HNRNPA1 is linked to diseases, including neurodegenerative disorders and cancer [161,162,163,164]. Emerging research has established the RNA-binding protein HNRNPA1 as a critical post-transcriptional regulator of metabolic homeostasis. Its influence extends across key metabolic tissues, most notably the liver and skeletal muscle, where it orchestrates the expression of a diverse network of target genes [165,166,167,168]. A recent study identified hnRNPA1 as a critical molecular link, translating local adipose tissue inflammation into systemic metabolic dysfunction [88]. HNRNPA1 expression is markedly reduced in white adipose tissue depots of obese human subjects and established murine models of obesity. The relationship is dynamic and potentially reversible, as demonstrated by the upregulation of adipose HNRNPA1 expression in obese individuals after undergoing therapeutic weight loss surgery. Researchers created adipocyte-specific HNRNPA1-knockout mice to establish direct causation by selectively deleting the gene in adipose tissue. This resulted in a significantly worsened metabolic syndrome, marked by severe conditions such as increased adipose tissue inflammation, fibrosis, profound systemic insulin resistance, and notable hepatic steatosis. HNRNPA1 plays a critical role in adipocytes by maintaining metabolic homeostasis, and its absence can lead to significant metabolic dysfunction. The study revealed that HNRNPA1 directly interacts with the messenger RNA (mRNA) of CCL2, a significant pro-inflammatory chemokine. This binding event facilitates the destabilization of the *CCL2* transcript, resulting in its swift degradation and restricting the synthesis of the CCL2 protein. In conditions of HNRNPA1 deficiency, as observed in obesity, the regulatory mechanism is disrupted, resulting in a significant enhancement of CCL2 protein synthesis and secretion by adipocytes. Inhibition of the CCL2 signaling pathway pharmacologically in mice effectively reduced adipose tissue inflammation and led to notable enhancements in overall glucose homeostasis. This finding supports the HNRNPA1-CCL2 axis as a key factor in obesity-related pathology and identifies it as a potential target for future therapeutic strategies.

### 3.9. HnRNPA2B1 (Heterogeneous Nuclear Ribonucleoprotein A2/B1)

Heterogeneous nuclear ribonucleoprotein A2/B1 (hnRNPA2B1) is a member of the hnRNP family of RNA-binding proteins that regulate multiple aspects of RNA metabolism, including pre-mRNA processing, alternative splicing, RNA stability, intracellular transport, and translation [169,170,171]. HnRNPA2B1 has emerged as a critical post-transcriptional regulator of adipose tissue biology, integrating metabolic and inflammatory signals across distinct adipose depots. hnRNPA2B1 is highly expressed in mouse primary brown preadipocytes, where it participates in prostaglandin E2 (PGE2)–E-prostanoid receptor 3 (EP3) signaling to stabilize zinc finger protein 410 (*Zfp410*) mRNA, a transcription factor that promotes brown adipocyte differentiation [172]. More recent work has expanded this role by demonstrating that hnRNPA2B1 is a key driver of thermogenic programming in adipocytes, particularly in beige adipocytes arising from white fat depots. Under conditions of hyperthermia or stress signaling, induction of heat-shock factor 1 (HSF1) enhances hnRNPA2B1 transcription in adipose tissue. HnRNPA2B1 subsequently binds to the 3′ untranslated regions (3′ UTRs) of essential thermogenic transcripts, including *Ucp1 and Ppargc1a* (encoding PGC-1α), thereby increasing their mRNA stability [89]. This post-transcriptional mechanism facilitates white adipose tissue beiging and activation of thermogenic pathways that elevate energy expenditure. Notably, loss of hnRNPA2B1 selectively impairs beige fat induction, while classical brown adipose tissue markers are less affected in certain contexts, suggesting depot- and lineage-specific regulatory functions. In parallel with its thermogenic role, hnRNPA2B1 contributes to adipose tissue inflammation, particularly in diet-induced obesity. HnRNPA2B1 expression is upregulated in epididymal white adipose tissue (eWAT) of high-fat-diet-induced obese mice, where it promotes a pro-inflammatory milieu by stabilizing mRNAs encoding key cytokines such as Tnfα, Il-6, and Il-1β in macrophages [90]. This activity drives macrophage polarization toward the pro-inflammatory M1 phenotype, exacerbating adipose inflammation and metabolic dysfunction. Consistent with this, mice haploinsufficient for Hnrnpa2b1 (A2B1 HET) are protected from high-fat-diet-induced obesity and display reduced M1 macrophage infiltration in eWAT. The inflammatory actions of hnRNPA2B1 extend to paracrine regulation of adipocytes, as conditioned media from macrophages with altered hnRNPA2B1 expression modulate preadipocyte proliferation and white fat expansion. Accordingly, A2B1 HET mice exhibit reduced white fat mass and smaller adipocytes under obesogenic conditions. Translational relevance is underscored by human studies showing that pioglitazone treatment reduces hnRNPA2B1 expression in subcutaneous adipose tissue of individuals with type 2 diabetes, with changes in hnRNPA2B1 expression significantly correlating with improvements in insulin sensitivity, lipid metabolism, glycemic control, and body composition [91]. Collectively, these findings position hnRNPA2B1 as a nodal post-transcriptional regulator linking thermogenic capacity, immune–adipocyte crosstalk, and metabolic homeostasis. Future research should focus on defining the cell-type-specific RNA interactome of hnRNPA2B1 in adipocytes versus immune cells, elucidating how upstream stress and metabolic signals dynamically modulate its activity, and determining whether selective targeting of hnRNPA2B1–RNA interactions can uncouple its beneficial thermogenic effects from its pro-inflammatory actions. Such studies may pave the way for novel therapeutic strategies aimed at enhancing adipose tissue plasticity and improving metabolic health in obesity and type 2 diabetes.

### 3.10. PSPC1 (Paraspeckle Component 1)

Paraspeckle component 1 (PSPC1) is an RNA-binding protein that plays a key role in the formation and function of paraspeckles, nuclear bodies involved in gene regulation and RNA processing. As a member of the drosophila behavior/human splicing (DBHS) protein family, PSPC1 interacts with other core paraspeckle proteins such as NONO and SFPQ to regulate nuclear retention of RNAs containing inverted repeats. PSPC1 plays a critical role in the nucleus by binding to specific RNA transcripts and retaining them within the paraspeckles. This process of nuclear retention can sequester target RNAs and associated proteins, thereby controlling their availability for translation or regulating alternative splicing [92,173,174,175]. Beyond its role in cellular differentiation and the stress response, PSPC1 is frequently dysregulated in human cancers, where its overexpression is linked to enhanced tumor growth, epithelial-to-mesenchymal transition, and cancer metastasis, highlighting its significance as a potential oncoprotein and therapeutic target [175,176,177,178]. Recent research has highlighted the importance of PSPC1 in regulating adipogenesis and adipocyte metabolism [92]. PSPC1 is a direct target of PPARγ, and its expression is markedly upregulated during the adipogenic differentiation of 10T1/2 and 3T3-L1 preadipocytes. In addition, PSPC1 expression is significantly reduced in the white adipose tissue of animal models of obesity. Complementary in vitro and in vivo analyses demonstrated that PSPC1 expression facilitated adipocyte-specific protein expression and adipogenesis. Deletion of PSPC1 in adipose tissue in vivo impaired adipocyte-specific gene expression and reduced adipose tissue mass and adipocyte size. Loss of PSPC1 expression in adipose tissue in vivo compromised adipocyte development and lipid storage and affected the development of diet-induced obesity and insulin resistance. Mechanistically, PSPC1 directly interacted with a number of adipose-specific mRNA transcripts via its two RRM domains. PSPC1 interacted with the RNA export factor *Ddx3x* and translocated from the nucleus to the cytoplasm during the course of differentiation, thereby enhancing the nuclear export of its target transcripts, including *Ebf1, Pparγ, Acsl1, Scd1*, and Cd36. These findings highlight a critical post-transcriptional checkpoint in adipogenesis, governed by PSPC1, which is required for proper RNA maturation and ultimately for healthy adipose tissue development. While these studies provided important insights into the role of PSPC1 in adipogenesis and adipose tissue metabolism, several critical questions still remained unresolved. For instance, the expression pattern of PSPC1 across different human adipose tissue depots has not yet been fully characterized. It also remained unclear how metabolic conditions, particularly obesity and associated metabolic dysfunctions, influenced PSPC1 expression levels and localization within these depots. Understanding these aspects would be essential to determine whether PSPC1 plays a conserved and clinically relevant role in regulating adipose tissue function and systemic metabolic homeostasis in humans.

### 3.11. RBMS1 (RNA-Binding Motif Single-Stranded Interacting Protein 1)

RNA-binding motif single-stranded interacting protein 1 (RBMS1) contains two conserved RNA recognition motifs (RRMs) located in its N-terminal region. Each RRM includes characteristic RNP1 and RNP2 consensus sequences that allow binding to single-stranded RNA and DNA. RBMS1 is a pivotal regulator of post-transcriptional gene expression, influencing essential cellular processes such as mRNA splicing, stability, and translation initiation. Dysregulation of RBMS1 has been implicated in oncogenesis, with aberrant expression patterns linked to tumor progression and metastasis across multiple cancer types [179,180]. Beyond cancer, RBMS1 has been associated with cardiovascular disease, potentially contributing to coronary heart disease through its involvement in lipid metabolism and inflammatory pathways [181,182]. Moreover, genetic studies have identified RBMS1 polymorphisms as risk factors for type 2 diabetes, underscoring its role in metabolic regulation [86,93,94]. Our recent findings further reveal a critical function for RBMS1 in adipogenesis and adipose tissue metabolism [95]. RBMS1 is abundantly expressed in 3T3-L1 preadipocytes but is downregulated during adipogenic differentiation. In mice subjected to a high-fat diet, RBMS1 expression is markedly elevated in both subcutaneous and visceral adipose tissues. Knockdown of RBMS1 in 3T3-L1 cells led to decreased expression of key adipogenic markers such as CEBPα and lipid metabolism enzymes ATGL and HSL. Notably, although *Atgl* mRNA levels remained constant, its protein abundance declined, suggesting that RBMS1 modulates ATGL expression through post-transcriptional mechanisms. Given ATGL’s central role in lipolysis, disruption of its regulation by RBMS1 could contribute to metabolic pathologies including obesity and diabetes. Transcriptomic and proteomic analyses further demonstrated that RBMS1 depletion alters a broad spectrum of genes and proteins involved in carbohydrate and lipid metabolism, highlighting its multifaceted role in metabolic homeostasis. While current findings establish RBMS1 as an important regulator of adipogenesis and adipocyte metabolism, further studies are needed to delineate its precise molecular mechanisms and physiological relevance. Future research should aim to identify the direct RNA targets of RBMS1 in adipocytes using approaches such as RNA immunoprecipitation sequencing (RIP-seq) or crosslinking immunoprecipitation (CLIP-seq) to define its post-transcriptional regulatory network. Additionally, conditional adipose-tissue-specific RBMS1-knockout mouse models would provide critical insights into its in vivo role in lipid storage, mobilization, and systemic energy homeostasis under normal and high-fat diet conditions. Proteomic analyses focusing on RBMS1-interacting proteins could uncover cofactors and signaling pathways that cooperate with RBMS1 to regulate translation and lipid metabolism. Furthermore, exploring the role of RBMS1 in human adipose tissue and assessing whether genetic variants or altered expression patterns correlate with obesity, insulin resistance, or type 2 diabetes could clarify its clinical significance.

### 3.12. MEX3C (Mex-3 RNA Binding Family Member C)

MEX3C is a protein-coding gene and a member of the MEX3 family of RNA-binding proteins. The name “MEX3” originates from the C. elegans protein Muscle EXcess-3, where it was first identified as a key regulator of embryonic development [183]. MEX3C, also known as RNF194 (Ring Finger Protein 194), is a conserved protein found in humans and other vertebrates that plays a critical role in post-transcriptional gene regulation. MEX3C is a bifunctional protein characterized by two key structural domains that enable distinct regulatory roles. Its two K-homology (KH) domains function as RNA-binding motifs, allowing MEX3C to selectively recognize and bind specific sequences within the 3′ untranslated regions (3′ UTRs) of target mRNAs, thereby influencing their stability and translation. In addition, the protein contains a RING finger domain that imparts E3 ubiquitin ligase activity, enabling it to tag target proteins with ubiquitin for proteasomal degradation. Through this dual mechanism, MEX3C simultaneously modulates gene expression at both the post-transcriptional and post-translational levels, integrating RNA and protein regulation within a single molecule. The MEX3C gene plays a critical role in regulating growth and energy metabolism in mice. Studies have shown that MEX3C mutation leads to growth retardation, primarily due to insulin-like growth factor 1 (IGF1) deficiency in developing bone tissue [184]. In addition to impaired growth, homozygous MEX3C mutant mice exhibit markedly reduced adiposity compared with age-matched wild-type controls. Both heterozygous and homozygous mutants show decreased adipose tissue deposition accompanied by enhanced energy expenditure, suggesting that MEX3C functions as a key regulator of energy balance. Further investigations revealed that homozygous MEX3C gene-trap mice display elevated physical activity, which likely contributes to their increased metabolic rate [96]. Importantly, MEX3C mutation provides full protection against diet-induced obesity (DIO) and its associated metabolic disturbances, including hyperglycemia, insulin resistance, hyperlipidemia, and hepatic steatosis. Even in leptin-deficient ob/ob mice, introduction of the MEX3C mutation led to improved glucose and lipid profiles and enhanced locomotor activity, reinforcing the idea that MEX3C influences systemic energy metabolism through central, possibly neuronal, mechanisms. Interestingly, our recent observations highlight a previously uncharacterized role of MEX3C in adipose tissue biology. We found that MEX3C is highly expressed in 3T3-L1 preadipocytes, but its expression declines significantly during adipogenic differentiation, implying a potential role in adipogenesis [97]. Moreover, MEX3C expression is markedly reduced in the adipose tissue of HFD-fed mice, a condition commonly associated with obesity-induced adipose dysfunction. These findings suggest that MEX3C may act as a molecular link between adipogenesis, energy metabolism, and obesity. Future research should aim to dissect the tissue-specific functions of MEX3C, particularly within adipose depots. The development of adipose-specific MEX3C-knockout mouse models would be invaluable to clarify whether MEX3C acts locally within adipocytes to influence lipid storage, thermogenesis, and energy homeostasis or whether its effects are mediated primarily through neural pathways.

### 3.13. PCBP2 (Poly(rC)-Binding Protein 2)

PCBP2 is an RNA-binding protein that recognizes poly(rC) sequences through its KH domains and plays a central role in post-transcriptional gene regulation. While early research primarily focused on its functions in viral and bacterial infections or in regulating globin mRNA, more recent studies have expanded its relevance to broader cellular contexts, linking PCBP2 to metabolic regulation and cell differentiation processes [185,186,187,188,189,190,191,192]. PCBP2 is expressed in adipose tissue, where it appears to play a functional role in regulating adipose-derived cell differentiation. According to the Human Protein Atlas, PCBP2 shows “mixed function” expression in adipose tissue, suggesting involvement in multiple cellular processes. Comparative transcriptomic analyses have shown that PCBP2 is differentially expressed in adipose-derived mesenchymal stromal/stem cells (AMSCs) compared to bone-marrow-derived MSCs (BMSCs), highlighting its potential contribution to tissue-specific lineage commitment. In the study by Gluscevic et al., PCBP2 was identified as a gene supporting adipogenic lineage commitment in AMSCs [98]. Functional experiments demonstrated that modulating PCBP2 expression influenced adipogenic differentiation but not osteogenic differentiation, indicating that PCBP2 helps direct AMSCs toward the adipocyte lineage and contributes to the distinct differentiation potential of adipose-derived cells. The mechanistic details of how PCBP2 influences adipogenic differentiation in adipose-derived MSCs need to be studied, e.g., what target mRNAs does PCBP2 bind in that context? Do those target mRNAs encode adipogenic transcription factors, lipid metabolism enzymes, or other modulators? Does PCBP2 play a role in mature adipocytes (white adipose tissue, brown adipose tissue) in vivo (in humans or animals) rather than just in in vitro MSC differentiation? The role of PCBP2 in the context of obesity, insulin resistance, adipose tissue inflammation or browning of adipose tissue remains unknown.

### 3.14. Sam68

**Figure 4:**
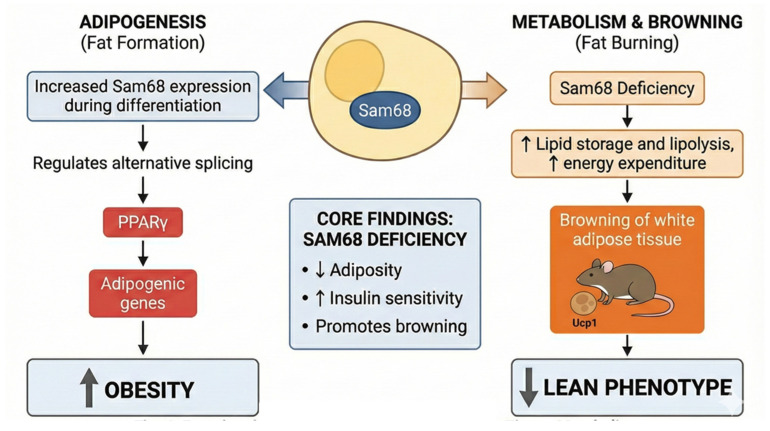
Functional significance of Sam68 in adipose tissue metabolism.

Sam68 (KHDRBS1) is a multifunctional RNA-binding protein that links cell signaling pathways to post-transcriptional gene regulation [193,194]. It contains a KH RNA-binding domain, proline-rich interaction motifs, and multiple phosphorylation sites that allow it to respond dynamically to kinase signaling. Functionally, Sam68 regulates alternative splicing, mRNA export, stability, and translation, enabling it to control key cellular processes such as cell cycle progression, differentiation, metabolism, and stress responses. Physiologically, Sam68 is involved in mitosis, fertility, and neuronal cells, and its dysregulation contributes to cancer and neurological disorders [195,196,197,198]. Sam68 is highly expressed in both white and brown adipose depots. Mouse genetic studies have shown that Sam68 expression in adipose tissue is functionally important during adipocyte lineage commitment and differentiation [99]. Whole-body Sam68-knockout mice display markedly reduced adipose mass (both WAT and BAT) and resistance to high-fat diet (HFD)-induced obesity, pointing to a role early in adipocyte development and in adipose physiology. During adipogenesis, Sam68 orchestrates alternative splicing of key components of the mTOR/S6K1 signaling axis, thereby sustaining mTORC1 activity required for adipogenic transcriptional programs. Loss of Sam68 disrupts these splicing events, impairs adipocyte differentiation, and reduces adipocyte formation. Sam68 also governs adipocyte progenitor number and commitment through broad splicing regulation of genes controlling cell-cycle progression, growth, and lineage specification, contributing to diminished adipose mass in knockout models [99]. Beyond development, Sam68 functions as a suppressor of thermogenic remodeling; its genetic ablation promotes browning of white adipose tissue, upregulates Ucp1, enhances whole-body energy expenditure, and confers resistance to diet-induced obesity [100,101]. In addition, SAM68 is directly involved in regulating insulin signaling in various cell types including adipocytes [99,102,103]. These diverse actions are shaped by post-translational modifications, particularly phosphorylation, that refine Sam68’s RNA-binding capacity and spliceosomal interactions, linking nutrient and hormonal cues to RNA processing. Collectively, these findings establish Sam68 as a central integrator of adipocyte development, thermogenic programming, and metabolic control, underscoring its potential as a therapeutic target for obesity and metabolic disease. The functional significance of Sam68 in adipose tissue metabolism is shown in Figure 4. Continued work defining its RNA targets, regulatory modifications, and human relevance will inform its potential as a metabolic therapeutic target.

## 4. Emerging Therapeutic Approaches to Target RNA-Binding Proteins

Extensive evidence demonstrates that dysregulation of RNA-binding proteins (RBPs), through altered expression levels, subcellular localization, or post-translational modification, plays a central role in the pathogenesis of diverse diseases, including cancer, neurodegenerative disorders, and metabolic dysfunctions [57,60,62,65]. Accordingly, RBPs have emerged as compelling therapeutic targets, driving increasing interest in pharmacological strategies aimed at modulating their function [199,200]. However, therapeutic targeting of RBPs has historically been challenging. Most RBPs lack well-defined hydrophobic pockets typically required for high-affinity binding of drug-like small molecules, and their interactions with RNA are often mediated by large, dynamic, and highly charged surfaces. Moreover, individual RBPs frequently regulate broad RNA networks, raising concerns regarding target selectivity and unintended perturbation of global RNA metabolism. These features have contributed to the longstanding perception of RBPs as “undruggable” targets [200]. Despite these challenges, substantial progress has been made in recent years through the development of agents that directly or indirectly modulate RBP activity, localization, or stability. Multiple small-molecule inhibitors have now been identified that disrupt RNA–RBP interactions, alter RBP subcellular trafficking, or promote protein destabilization. Among the most extensively studied examples is the RNA-binding protein HuR (ELAVL1), a key post-transcriptional regulator of oncogenic gene expression. Several HuR inhibitors have been developed and evaluated in preclinical cancer models. MS-444 is one of the best-characterized HuR inhibitors and acts primarily by disrupting HuR homodimerization, a process required for stable RNA binding and cytoplasmic accumulation [201,202,203,204]. MS-444 binds to the RNA recognition motif (RRM) region of HuR, preventing its nuclear export and thereby limiting HuR-mediated stabilization of oncogenic mRNAs. Consequently, MS-444 reduces the expression of HuR targets such as COX-2, cyclin D1, and VEGF, leading to impaired tumor cell proliferation, induction of apoptosis, and suppression of tumor growth in colorectal and pancreatic cancer models. Additional HuR-directed compounds further illustrate the feasibility of targeting RNA–protein interactions. CMLD-2, identified through structure-guided screening, directly inhibits HuR–RNA binding by occupying the RNA-binding surface of HuR and blocking its association with AU-rich elements in target transcripts. Functionally, CMLD-2 reduces cancer cell viability, migration, and invasion and suppresses tumor growth in preclinical models of breast and thyroid cancer [205,206]. KH-3 is another synthetic HuR inhibitor that selectively disrupts HuR–ARE interactions without significantly altering HuR protein abundance. This inhibition destabilizes oncogenic transcripts such as XIAP and cyclin E1, promoting apoptosis and enhancing chemosensitivity in solid tumor models, thereby supporting its potential use in combination therapies [207,208,209]. Beyond HuR, several other RBPs have been successfully targeted using small-molecule inhibitors in preclinical studies. These include IGF2BP1 (inhibited by BTYNB), hnRNPA1 (targeted by VPC-80051), and Musashi proteins (targeted by Ro 08-2750), collectively demonstrating that diverse RBP families are amenable to pharmacological modulation [210,211,212,213,214]. In parallel, nucleic-acid-based approaches such as antisense oligonucleotides (ASOs) and RNA interference (RNAi) have been employed to suppress RBP expression at the mRNA level. ASOs targeting eIF4E, LIN28B, and Musashi-2 (MSI2) have shown robust anti-tumor activity in preclinical cancer models, particularly in leukemia and aggressive solid tumors, although challenges related to delivery, stability, and tissue specificity remain [215,216]. More recently, targeted protein degradation has emerged as a promising strategy for RBP modulation. Proteolysis-targeting chimeras (PROTACs) designed to selectively degrade oncogenic RBPs, including LIN28 and members of the IGF2BP family, offer the potential for more durable suppression of pathogenic RNA regulatory programs by eliminating the target protein rather than merely inhibiting its activity [217]. Collectively, these advances underscore the growing translational potential of RBPs as drug targets in cancer. Importantly, as our understanding of RBP biology in adipose tissue continues to expand, these therapeutic strategies may extend beyond oncology. RBPs are increasingly recognized as critical regulators of adipocyte differentiation, lipid metabolism, inflammation, and insulin sensitivity. Thus, RBP-targeting agents developed in the context of cancer may represent a novel therapeutic avenue for metabolic disorders such as obesity and type 2 diabetes, particularly in disease states driven by aberrant post-transcriptional regulation. Continued advances in structural biology, chemical biology, and RNA-based therapeutics are expected to further accelerate the development of clinically viable RBP-directed therapies across multiple disease contexts.

## 5. Conclusions

**Figure 5:**
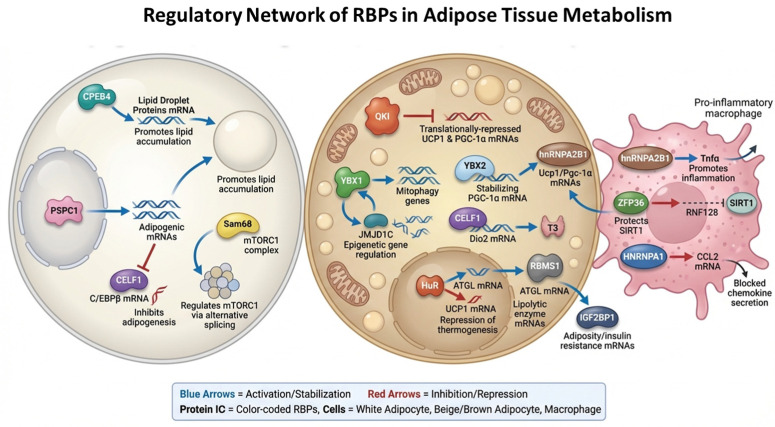
Regulatory functions of RNA-binding proteins in adipose tissue metabolism.

The functional significance of RNA-binding proteins (RBPs) in adipose tissue is increasingly recognized as profound, given their central roles in regulating post-transcriptional gene expression, adipocyte differentiation, lipid metabolism, and inflammatory responses (illustrated in Figure 5).

Consequently, RBPs represent promising and largely untapped therapeutic targets for alleviating adipose tissue dysfunction and obesity-associated metabolic disorders such as insulin resistance, type 2 diabetes, and cardiovascular disease. However, to fully harness their therapeutic potential, several critical questions must be addressed. First, to date, only a limited number of RBPs have been functionally characterized within the context of adipose tissue biology, while the vast majority remain largely unexamined. This significant knowledge gap limits our understanding of how post-transcriptional regulation shapes adipose tissue development, metabolic adaptation, and dysfunction under obese conditions. Through a combination of integrative bioinformatic analyses and targeted experimental validation, we have recently uncovered a subset of RBPs that exhibit distinctive and condition-dependent expression patterns in adipose tissue. These RBPs display dynamic regulation across different adipose depots and metabolic states, suggesting potential roles in key processes such as adipocyte differentiation, lipid storage, thermogenesis, and inflammatory signaling. Their expression signatures in obesity and metabolic stress further implicate them as potential modulators of adipose tissue plasticity and systemic energy homeostasis. Consequently, functional analysis of these RBPs is a necessary and promising next step. Second, given the dynamic cellular heterogeneity of adipose tissue, particularly under obese conditions, future research should integrate single-cell transcriptomic and spatial profiling approaches to unravel the cell-type-specific expression patterns and regulatory roles of RNA-binding proteins (RBPs). Obesity profoundly remodels adipose tissue composition, altering the proportions of adipocytes, preadipocytes, immune cells, and stromal populations. These shifts can mask gene expression signals in bulk tissue analyses, making it difficult to discern the true cellular sources and regulatory targets of RBPs. Employing single-cell RNA sequencing (scRNA-seq) and spatial transcriptomics would enable precise mapping of RBP expression across diverse adipose cell subtypes and microenvironments, clarifying how RBP activity is modulated within specific populations such as hypertrophic adipocytes or pro-inflammatory macrophages. Ultimately, these high-resolution approaches could pinpoint the cellular contexts in which RBPs exert their metabolic functions, paving the way for more targeted and effective therapeutic strategies. Third, it is essential to investigate how RBP expression and activity are influenced by metabolic and hormonal cues, including insulin, catecholamines, glucagon, and nutrient availability. Understanding these regulatory interactions could elucidate how RBPs integrate into broader metabolic networks that govern energy balance. For example, if RBPs respond dynamically to fluctuations in glucose or lipid levels or to endocrine signals controlling nutrient sensing, they may act as molecular bridges connecting metabolic status to post-transcriptional gene regulation and cellular adaptation. Additionally, emerging evidence highlights that small biomolecules (SBMs), such as sugars, nucleotides, metabolites like S-adenosylmethionine (SAM) and NAD(P)H, and even certain drugs, can directly bind to RBPs, altering their conformation, localization, and RNA-binding activity. These context- and concentration-dependent interactions link RBP function tightly to cellular metabolic states. Exploring this layer of regulation within adipose tissue could uncover novel mechanisms by which metabolic homeostasis is maintained or disrupted under physiological and pathological conditions. Finally, translating RBP biology into therapeutic applications will require the development of specific small-molecule inhibitors or activators through rational drug design. Such compounds could be evaluated in animal models of obesity to assess their efficacy in modulating metabolic pathways, improving insulin sensitivity, reducing adiposity, and restoring overall metabolic balance. Promising preclinical outcomes could ultimately pave the way for clinical trials and the development of innovative RBP-targeted therapies for obesity and related metabolic diseases.

## Figures and Tables

**Table 1:** RNA-binding proteins and their established roles in adipose tissue metabolism. RRM—RNA recognition motif; CSD–cold-shock domain; KH domain—K-homology domain; Znf—zinc fingers.

RBP Name	Binding Domain	Target RNAs	Functional Significance	References
QKI	KHx1	*Ucp1*, *Pgcα*	Negative regulator of thermogenesis	[66]
HuR	RRMx3	*Atgl*, *Insig1*	Negative regulator of adipogenesis, thermogenesis, and lipid homeostasis	[67,68,69,70,71,72]
YBX-1	CSD	*Pink1 Ulk1* *Jmjd1c*	Promotes thermogenesis and adipogenesis	[73,74,75,76]
YBX-2	CSD	*Cidec and Plin1*	Regulator of adipogenesis and lipid storage	[77,78]
CELF1	RRMx3	*C/Ebpβ*, *Dio2*	Inhibits adipogenesis, activates thermogenesis, and promotes energy expenditure	[79,80]
IGF2BP1	RRMx2; KHx4	Not known	Regulates adipogenesis and adipose metabolism	[81,82,83]
ZFP36	Tandem CCCH zinc-finger domains	*Fgf21*, *Rnf128*	Lipid metabolism and whole-body insulin function	[84,85]
CPEB4	RRMx2	Not known	Pro-adipogenic factor, and its inhibition protects against obesity and metabolic disease	[86,87]
HnRNPA1	RRMx2	*Ccl2*	Regulates metabolic homeostasis by reducing adipose tissue inflammation	[88]
HnRNPA2B1	RRMx2	Tnfα, Il-6, and Il-1β	Cold induced thermogenesis, inflammation	[89,90,91]
PSPC1	RRMx2	*Ddx3x*	Adipogenesis and lipid storage	[92]
RBMS1	RRMx2	Not known	Regulates adipogenic differentiation	[93,94,95]
MEX3C	KHx2; Znf_RINGx1	Not known	Whole-body energy metabolism	[96,97]
PCBP2	KHx3	Not known	Adipogenesis	[98]
SAM68	KHx1	Not known	Adipogenesis, thermogenesis, and insulin signaling	[99,100,101,102,103]

## Data Availability

No new data were created or analyzed in this study. Data sharing is not applicable to this article.

## Abbreviations

The following abbreviations are used in this manuscript:

RBP	RNA-binding protein
QKI	Quaking
WAT	White adipose tissue
BAT	Brown adipose tissue
PPARγ	Peroxisome proliferator-activated receptor gamma
HuR	Human antigen R
BMI	Body mass index
